# Role of Interferon Gamma Release Assay in Active TB Diagnosis among HIV Infected Individuals

**DOI:** 10.1371/journal.pone.0005718

**Published:** 2009-05-28

**Authors:** Basirudeen Syed Ahamed Kabeer, Rajasekaran Sikhamani, Sowmya Swaminathan, Venkatesan Perumal, Paulkumaran Paramasivam, Alamelu Raja

**Affiliations:** 1 Department of Immunology, Tuberculosis Research Centre (ICMR), Tamil Nadu, India; 2 Division of HIV/AIDS, Tuberculosis Research Centre (ICMR), Tamil Nadu, India; 3 Department of Statistics, Tuberculosis Research Centre (ICMR), Tamil Nadu, India; 4 Depatment of Clinic, Tuberculosis Research Centre (ICMR), Tamil Nadu, India; 5 Government Hospital of Thoracic Medicine, Tambaram Sanatorium, Tambaram, Tamil Nadu, India; McGill University, Canada

## Abstract

**Background:**

A rapid and specific test is urgently needed for tuberculosis (TB) diagnosis especially among human immunodeficiency virus (HIV) infected individuals. In this study, we assessed the sensitivity of Interferon gamma release assay (IGRA) in active tuberculosis patients who were positive for HIV infection and compared it with that of tuberculin skin test (TST).

**Methodology/Principal Findings:**

A total of 105 HIV-TB patients who were naïve for anti tuberculosis and anti retroviral therapy were included for this study out of which 53 (50%) were culture positive. Of 105 tested, QuantiFERON-TB Gold in-tube (QFT-G) was positive in 65% (95% CI: 56% to 74%), negative in 18% (95% CI: 11% to 25%) and indeterminate in 17% (95% CI: 10% to 24%) of patients. The sensitivity of QFT-G remained similar in pulmonary TB and extra-pulmonary TB patients. The QFT-G positivity was not affected by low CD4 count, but it often gave indeterminate results especially in individuals with CD4 count <200 cells/µl. All of the QFT-G indeterminate patients whose sputum culture were positive, showed ≤0.25 IU/ml of IFN-γ response to phytohemagglutinin (PHA). TST was performed in all the 105 patients and yielded the sensitivity of 31% (95% CI: 40% to 22%). All the TST positives were QFT-G positives. The sensitivity of TST was decreased, when CD4 cell counts declined.

**Conclusions/Significance:**

Our study shows neither QFT-G alone or in combination with TST can be used to exclude the suspicion of active TB disease. However, unlike TST, QFT-G yielded fewer false negative results even in individuals with low CD4 count. The low PHA cut-off point for indeterminate results suggested in this study (≤0.25 IU/ml) may improve the proportion of valid QFT-G results.

## Introduction

Tuberculosis (TB) remains the single infectious disease, causing the highest mortality in humans, leading to 3 million deaths annually. Approximately 8–10 million people are infected with this pathogen every year [1]. The vast majority of TB cases are reported in Africa, South East Asia and Western Pacific countries. The recent increase in the number of cases even in developed countries, associated with the spread of human immunodeficiency virus (HIV) infection, has had a major impact on the current situation of TB. India accounts for a huge number of HIV-TB cases and ranks first in the world in terms of incident of TB cases. [2].

Individuals with HIV infection are at increased risk of rapid progression of a recently acquired tuberculous infection, as well as of re-activation of latent TB infection (LTBI). Delayed diagnosis of TB and initiation of appropriate treatment more than 3 weeks after presentation, are associated with 45–85% of deaths in HIV infected patients [3]. Early diagnosis and prompt treatment for TB are the key elements to control the mortality rate of HIV infected subjects. The clinical features of HIV-infected patients with TB are often non-specific. Decreased tuberculin reactivity, lower sensitivity of acid fast staining, atypical radiographic presentations, and similarity in presentation with other HIV related infections hinder the diagnosis of TB in HIV infected patients [4].

Recently introduced Interferon gamma release assays (IGRA) are promising tests for the diagnosis of TB infection. IGRA is available in two commercial formats: (i) Quantiferon TB Gold (QFT-G) (Cellestis Ltd, Victoria, Australia), which measures the quantity of IFN-γ secreted by T cells and (ii) T-Spot.TB assay (Oxford Immunotech, Oxford, UK) which enumerates the number of IFN-γ secreting T cells after *in vitro* stimulation with TB-specific antigens. T-Spot.TB assay uses only Early Secreted Antigen Target (ESAT)-6 and Culture filtrate protein (CFP)-10, whereas an additional antigen TB7.7 is incorporated in Quantiferon TB Gold. Several studies have been conducted in various clinical settings on the accuracy and utility of IGRA and these have been reviewed elsewhere [5]. Most of these studies have reported that sensitivity of IGRA is modest to detect active TB disease [6]–[12] and also suggested that IGRA alone [13], [14] or in combination with TST [15] can be used to exclude the suspicion of active TB disease. Nevertheless, in general there is a concern about the sensitivity of IGRA in immunosuppressed (especially HIV) patients, as these assays are T-cell based whose number is often compromised in these patients [16]. Only a very few studies have been conducted on IGRA assays in immunocompromised patients, especially from TB endemic countries [17], [18], emphasizing the need for more such studies to test the validity of the IGRA assays in such settings. Hence, in this study, we aimed to measure the sensitivity of IGRA in diagnosing of active TB among HIV positive subjects in a country like India where both the diseases are co-existing and are endemic.

## Materials and Methods

This study was approved by the Scientific Advisory Committee and Institutional Ethical Committee of Tuberculosis Research Centre, Chennai. A written and informed consent was obtained from all the study participants before drawing blood.

### Study subjects recruitment

The recruitment of study subjects were done at Government Hospital of Thoracic Medicine, Tambaram, Chennai during April 2007 and March 2008. The demographic details and information on previous tuberculin skin test (TST) results were collected. Individuals with previous history of TB, silicosis, end stage renal disease, leukemia/lymphoma, who had TST in the past 16 months, under ATT for more than two weeks or ART or immunosuppressive therapy were excluded from the study. Pregnant and lactating patients were also excluded.

After registering the eligible patients, the radiological examination was carried out. A total of six sputum samples were collected from each study subject and they were stained for acid fast bacilli (AFB) microscopy. The staining for acid fast bacilli was done by Ziehl-Neelsen method [19]. Three sputum samples of each subject were cultured in conventional Lowenstein Jensen (Biomerieux Inc., Marcy I'Etoile, France) and also in liquid MP BacT medium (Biomerieux Inc., Marcy I'Etoile, France). The presence of *M. tuberculosis* in the positive culture samples was further confirmed by Gen-probe based PCR (Biomerieux Inc, Marcy I'Etoile, France) method.

The presence of active TB was defined as positive for sputum smear microscopy and/or identification of *M. tuberculosis* in sputum culture and/or abnormality suggestive of TB in chest x-ray. In addition, Fine Needle Aspiration Cytology (FNAC) was carried out in the individuals with extra pulmonary manifestations who were clinically suspected but had negative sputum results and normal chest x-ray. The diagnosis of pulmonary TB for the individuals with culture and smear negativity was based on chest x-ray finding and the clinician's opinion based on the clinical manifestations.

Blood was drawn from all the HIV-TB patients for total blood count, HIV serology and QFT-G. Then the TST was carried out. After the diagnosis, the patients were referred to respective treatment centers.

### HIV testing

The HIV status was confirmed by 2 rapid tests (Retroquic Comb Aids-RS, Span Diagnostics, India and HIV TRI-DOT, J. Mitra & Co, India). When a serum was positive for both tests, it was considered as HIV positive. If a serum was positive for only one EIA (which was rare), Western Blot was done as confirmatory test.

### CD4 count

The CD4 cell count was estimated in blood samples of HIV positive individuals by flow cytometry. 100 µl of whole blood was labelled with saturating concentrations of anti CD3-FITC, anti CD4-PE and anti CD8-APC (BD Biosciences, CA, USA). After 30 min incubation at 4°C, the red blood cells were lyzed using FACS lysing solution (BD Biosciences, CA, USA) and then fixed with 1% paraformaldehyde (Sigma Chemicals Co., MO, USA). The acquisition was done on FACS Calibur (BD Biosciences, CA, USA) and the percentages of CD3, CD4 and CD8 cells among the total lymphocytes were obtained using Flowjo Software (Tree star, Inc., CA, USA). The absolute CD3, CD4 and CD8 counts were calculated by multiplying the percentage with the total lymphocyte count.

### Interferon gamma release assay

The IFN-γ release assay was performed using Quantiferon TB-Gold In-tube (QFT-G) test (Cellestis Ltd., Victoria, Australia). One ml of blood was taken in each of the three tubes precoated with TB–antigen, phytohemaglutinin (PHA) for the positive control or no antigen for the negative control. The blood samples were drawn between 10 and 11 AM and taken to the lab within 2 hrs of phlebotomy. The tubes were incubated for 16–24 hrs at 37°C and plasma were collected after centrifugation and stored at 4°C until assayed. Within two weeks of time, QFT-G enzyme linked immunosorbant assay (ELISA) was carried out. The test results were interpreted using the software given by the manufacturer (Cellestis Ltd., Victoria, Australia) and the cut-off point for the diagnosis was determined as per manufacturer's instructions. If the IFN-γ secretion in response to TB antigen was ≥0.35 IU/ml, after subtracting NIL control IFN-γ, it was considered as positive for QFT-G and if the value was <0.35 IU/ml, it was considered as negative. If the negativity was associated with poor PHA response (i.e. IFN-γ secretion in response to PHA, after subtracting NIL control IFN-γ was <0.5 IU/ml), it was considered as indeterminate or invalid result for QFT-G.

### Tuberculin Skin Test

The 2 TU (tuberculin unit) of purified protein derivative (PPD) RT23 (Staten Serum Institute, Copenhagen, Denmark) was injected intradermally by Mantoux method and the induration was measured between 48–72 hrs after PPD injection by trained professionals. The cut-off point for TST positivity was considered as 5 mm for this study [20], [21].

### Statistical Analysis

Data were analyzed using SPSS 15.0 and Graphpad Prism 4.0 software. Mann-Whitney “U” test was carried out to calculate the difference between the groups. Logistic regression analysis was performed to examine the effect of potential variables on the odds of presenting a positive QFT-G test response. Odds ratios (OR) and their 95% confidence intervals (CI) were estimated by multivariate analyses. In these analyses, continuous variables were taken as dichotomous variables using the following arbitrary cut-off values: Age-36 years (median of the study population) and BMI-18.5 kg.m^2^. Based on the CD4 count, the patients were classified as <50 (Advance stage), 50–199 (AIDS defining) and >200 cell/µl to assess QFT-G and TST results.

## Results

A total of 300 HIV positive subjects were assessed during the study period. Among them 112 (37%) were identified as HIV-TB positive patients. 7 (6%) out of 112 patients refused to participate in this study and hence the remaining 105 were included. Among 105 patients who all were naïve for ATT and ART, 53 (50%) were culture confirmed TB patients. The demographic and baseline characteristics of all the 105 study subjects are given in **Table 1**.

**Table 1:** Demographic and baseline parameters of all the 105 HIV-TB patients.

Category	Subcategory	N (%) or Median (Range; IQR)
Sex, Number (%)	Male	84 (80)
	Female	21 (20)
Age, Median in years (Range; IQR)		36 (18–63; 25, 36)
BMI, kg/m^2^ Median (Range; IQR)		19 (13–31; 17,21)
HIV strain, Number (%)	HIV-I	94 (90%)
	HIV-I&II	11 (10%)
TB types, Number (%)	PTB	66 (63%)
	EPTB	39 (37%)
Smear, Number (%)	Positives	47 (45%)
	Negatives	58 (55%)
Culture, Number (%)	Positives	53 (50%)
	Negatives	52 (50%)
CD4, Median (Range; IQR) (Available for 81 subjects)		116 (11–2062; 48, 209)

BMI – Body mass index.

PTB – Pulmonary tuberculosis.

EPTB- Extra-pulmonary tuberculosis.

IQR-Inter quartile range.

### Sensitivity of QFT-G and TST

**Figure 1:**
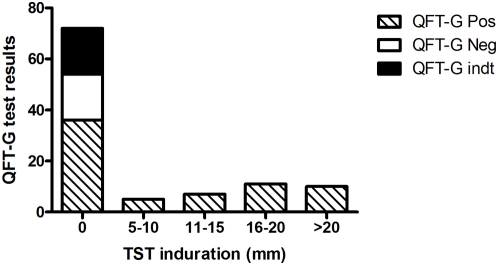
Agreement between TST and QFT-G test results. Among the 105 subjects tested, 72 were negative for TST. Of the 72 TST negative, 50% were positive for QFT-G. None of the TST positive was negative or indeterminate for QFT-G. TST-Tuberculin Skin Test, QFT-G-Quantiferon TB Gold in-tube, Pos - Positive, Neg - Negative, Indt – Indeterminate.

The results of QFT-G and TST are given in **Table 2**. Of 105 patients tested, 68 (65%, 95% CI: 56% to 74%) were positive and 19 (18%, 95% CI: 11% to 25%) were negative for QFT-G. The remaining 18 (17%, 95% CI: 10% to 24%) patients showed indeterminate results. All the indeterminate or invalid results were due to poor response to PHA. Among the 105 patients, 33 showed ≥5 mm induration for TST yielding the sensitivity of 31% (95% CI: 22% to 40%). When comparing QFT-G and TST results, the former was 33% more sensitive than the latter (P<0.001). None of the TST positives were negative or indeterminate for QFT-G (**Figure 1**). Among the 72 TST negatives, 50% were positive and 25% were negative for QFT-G.

**Table 2:** Performance of QFT-G and TST in subgroups of HIV-TB patients.

Groups	QFT-G			TST	
	% Pos (95%CI))	% Neg (95%CI)	% Indt (95%CI)	% Pos (95%CI)	% Neg (95%CI))
**Overall (N = 105)**	65 (56–74)	18 (11–25)	17 (10–24)	31 (22–40)	69 (60–78)
**PTB (N = 66)**	59 (47–71)	20 (10–30)	21 (11–31)	26 (15–37)	74 (63–85)
**Sputum culture positive (N = 44)**	66 (52–80)	11 (2–20)	23 (11–35)	25 (12–38)	75 (62–88)
**Sputum culture negative (N = 22)**	45 (24–66)	37 (17–57)	8 (2–34)	27 (8–46)	73 (54–92)
**Sputum smear positive (N = 43)**	68 (54–82)	9 (1–18)	23 (10–36)	26 (13–39)	74 (61–87)
**Sputum Smear negative (N = 23)**	44 (24–64)	39 (19–59)	17 (2–32)	26 (8–44)	74 (56–92)
**EPTB (N = 39)**	77 (64–90)	13 (2–24)	10 (1–19)	41 (25–56)	59 (44–74)

Pos – Positive.

Neg – Negative.

Indt – Indeterminate.

Sen - Sensitivity.

PTB – Pulmonary tuberculosis.

EPTB- Extra-pulmonary tuberculosis.

In a sensitivity analysis to investigate whether inclusion of highly probable cases (which was diagnosed based on abnormalities found in the chest X-ray and clinician's opinion) affected the sensitivity of the tests, we calculated the sensitivity in culture positive and negative cases separately. The positivity obtained in culture positive PTB, culture negative PTB and EPTB patients were 66% (95% CI: 52%–80%), 45% (95% CI: 24% to 66%) and 77% (95% CI: 64%–90%) for QFT-G and 25% (95% CI: 12%–38%), 27% (95% CI: 8%–46%) and 41% (95%CI: 26%–56%) for TST respectively. The positivity of QFT-G was significantly higher than TST in culture positive PTB and EPTB groups (P<0.001 and P = 0.002 respectively) but not in culture negative PTB group (P = 0.347). The proportion of QFT-G indeterminate results obtained in these groups was 23%, 18% and 10% respectively.

### Influence of Immunosupression on QFT-G and TST

**Figure 2:**
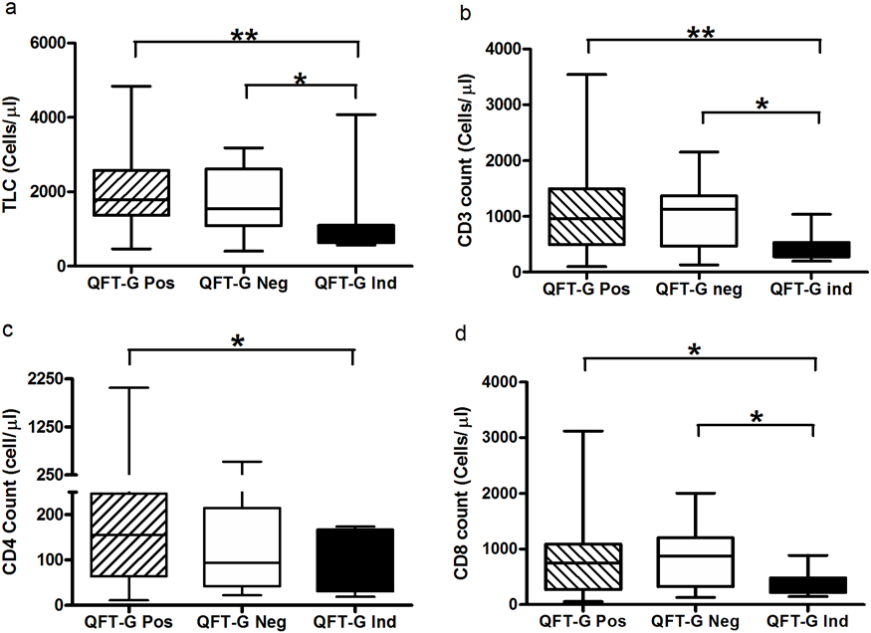
The level of total lymphocyte and T cell counts in QFT-G positive, negative and indeterminate subjects. The decline of total lymphocytes (a) or CD3 count (b) or CD4 count (c) or CD8 count (d) was associated with QFT-G indeterminate only but not with QFT-G negative. Box and Whisker plots show range, inter-quartile range and median. QFT-G – Quantiferon TB Gold (in-tube), * significant difference p<0.05, **significant difference p<0.01 by Mann-Whitney U test.

**Figure 3:**
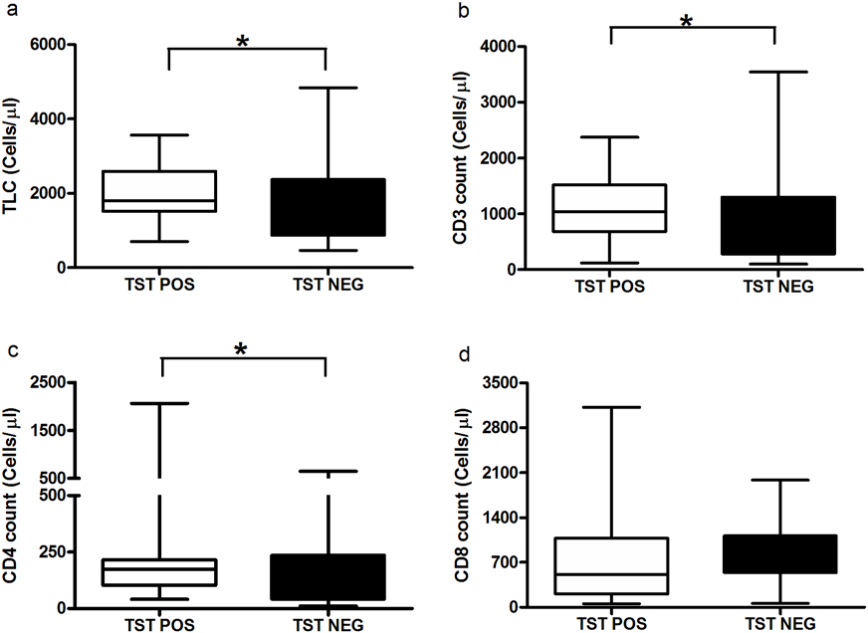
The level of total lymphocyte and T cell count in TST Positive and negative subjects. The level of total lymphocytes (a), CD3 count (b), CD4 count (c) were significantly low in TST negatives than TST positives whereas CD8 count (d) was not significantly different. Box and Whisker plots show range, inter-quartile range and median. TST – Tuberculin skin test, * significant difference p<0.05 by Mann-Whitney U test.

The median total lymphocyte, CD3, CD4 and CD8 counts did not differ significantly between QFT-G positive and negative subjects but was significantly lower in indeterminate subjects when compared to QFT-G positives (P = 0.0013, P = 0.003, P = 0.0418 and P = 0.0221 respectively) (**Figure 2**). When comparing the TST positive and negative subjects, the cell counts were significantly lower in TST negative than positive subjects (p = 0.0403, p = 0.0344 and P = 0.017 respectively) (**Figure 3**).

Further to confirm the influence of CD4 count on the positivity of QFT-G and TST, we developed a logistic regression model based on the CD4 counts (**Table 3**), for which, we stratified the CD4 cells counts as <50 cells/µl, 51–200 cells/µl and >200 cells/µl. When the QFT-G indeterminate results were considered as negative, the sensitivity of the assay was impaired in subjects with CD4 count <200 cells/µl (P = 0.047). This trend was not observed, if the indeterminate results were excluded (P = 0.124). TST positivity was decreased when the CD4 cell count dropped to <200 cells/µl, the (P = 0.04) (**Table 4**). The other tested parameters HIV strain, BMI, age, sex and types of TB were not found to influence the performance of QFT-G and TST.

**Table 3:** Analysis of variables associated with positive QFT-G test results.

Variables	With Indeterminate results			Without Indeterminate results		
	N	Odds Ratio (95%CI)	P	N	Odds Ratio (95%CI)	P
CD4 ≥200	81	7.058 (1.196–41.656)	0.047	70	4.596 (0.658–32.095)	0.124
Sex female	105	0.423 (0.107–1.673)	0.220	87	0.712 (0.147–3.456)	0.673
Age >36	105	1.282 (0.392–4.196)	0.681	87	0.960 (0.222–4.145)	0.956
BMI >18.5	91	1.005 (0.293–3.441)	0.994	78	1.890 (0.448–7.972)	0.386
EPTB	105	3.066 (0.765–12.284)	0.114	87	2.567 (0.564–11.691)	0.223
HIV strain I	105	1.295 (0.239–7.014)	0.764	87	0.836 (0.118–5.916)	0.858
Presence of Oral candidiasis	84	0.628 (0.150–2.625)	0.628	81	1.174 (0.203–6.783)	0.858

BMI – Body mass index.

N – Number of patients tested.

**Table 4:** Analysis of variance associated with TST positive results.

Variables	N	Odd Ratio (95% CI)	P
CD4 >200 cells/µl	81	10.881 (1.010–117.277)	0.04
Sex female	105	0.412 (0.084–2.032)	0.276
Age>36 years	105	2.226 (0.603–8.210)	0.230
BMI>18.5	91	0.740 (0.204–2.686)	0.647
EPTB	105	1.263 (0.357–4.464)	0.717
HIV strain I	105	2.079 (0.430–10.051)	0.363
Presence of Oral candidiasis	84	1.112 (0.294–4.212)	0.876

BMI – Body mass index.

N – Number of patients tested.

### QFT-G cut-off values

We analyzed the cut-off values for secretion of IFN-γ in response to TB antigens and PHA in culture confirmed TB cases. Applying the cut-off ≥0.13 IU/ml for TB antigens as suggested by Harada *et al* [22] to our data, did not improve the sensitivity of QFT-G. Only one indeterminate subject became positive for QFT-G.

A total of 36 subjects showed IFN-γ secretion <0.35 IU/ml in response to TB antigens. Among them 18 subjects were negative (i.e. IFN-γ secretion to PHA was >0.5 IU/ml) and the remaining 18 were indeterminate (i.e. IFN-γ secretion to PHA was <0.5 IU/ml) for QFT-G. The ranges of IFN-γ levels to PHA were 0.5 to 15.23 IU/ml in QFT-G negative subjects and 0–0.39 IU/ml in QFT-G indeterminate subjects. Of 18 indeterminate subjects, 12 (67%) showed ≤0.1 IU/ml IFN-γ response and 16 (89%) showed <0.25 IU/ml IFN-γ response to PHA (**Table 5**). Interestingly, all the culture positive HIV-TB cases who were indeterminate for QFT-G showed <0.25 IU/ml IFN-γ response to PHA.

**Table 5:** The level of IFN-γ secretion in response to PHA in QFT-G indeterminate subjects.

Subjects	Sputum culture results	Mitogen – Nil (IU/ml)
1	Negative	0
2	Positive	0.089
3	Positive	0.356
4	Positive	0
5	Negative	0.095
6	Positive	0.073
7	Negative	0.099
8	Negative	0.16
9	Negative	0.121
10	Positive	0.073
11	Positive	0.24
12	Positive	0.021
13	Positive	0.045
14	Positive	0.008
15	Negative	0.1
16	Negative	0.39
17	Positive	0.24
18	Positive	0.06

## Discussion

Early diagnosis of the active tuberculosis disease will help in earlier treatment, which is especially needed in HIV infected cases, since there is an accelerated progression of TB and higher mortality. However, there are lacunae in existing methods to diagnose TB in HIV positive subjects, which urge the development of alternative, rapid and accurate method. In this study, we have assessed the sensitivity of an emerging diagnostic method, IGRA among newly diagnosed HIV-TB patients.

QFT-G (In-tube) was recently approved by US Food and Drug Administration (FDA) in October 2007 and is the only commercially available IGRA test in India. Hence, in this study we used QFT-G (in-tube) method to analyze the role of IGRA.

In this study, we found that QFT-G can detect only 65% of active cases among the HIV infected individuals. Even in the culture confirmed TB cases, the QFT-G sensitivity was maintained as 66%. All the TST positives were positive for QFT-G. Hence, combining TST with QFT-G also could not enhance the overall sensitivity. If the indeterminate results were excluded, QFT-G showed 78% overall sensitivity and 88% sensitivity in culture confirmed cases. Hence, we conclude that QFT-G alone or in combination with TST cannot be used to exclude the active TB disease among HIV infected individuals. Of note, due to the inefficiency in discriminating the active and latent TB, the specificity of QFT-G and TST will always be low for active TB diagnosis in endemic countries like India (5).

The major concern in using T-cell based assay is the influence of CD4 count on the sensitivity. In QFT-G, the overlapping peptides of ESAT-6, CFP-10 and TB7.7 antigens are used, which are MHC class II restricted and largely recognized by CD4 T cells. Hence, it can be presumed that the sensitivity of QFT-G would be affected when CD4 count drops severely. While some of the studies showed IGRA was less influenced by HIV infection [23], [24], other study results contradict the observations [25], [18]. However, in our study we did not find any significant difference in CD4 count between QFT-G positive and negative subjects. The logistic regression model also confirmed that QFT-G negative results are CD4 count independent.

Another drawback of T-cell based assays is its poor sensitivity in EPTB patients. It was reported that in EPTB patients, the secretion of IFN-γ to *M. tuberculosis* antigens is poorer than the PTB patients [26]. Lee *et al* [27] reported that QFT-G is poor in detecting EPTB cases. Dewan *et al* [28] have shown only 16% sensitivity for QFT-G in EPTB patients. In contrast, we obtained 77% sensitivity using QFT-G in EPTB patients, in our study. Exclusion of indeterminate results yielded 86% sensitivity, which is similar to what we observed with culture confirmed PTB (88%). Hence, we conclude that the sensitivity of QFT-G is not compromised in EPTB when compared to PTB patients. However, since most of the EPTB subjects in our study group were TB lymphadenitis cases, further evidence is needed on the sensitivity of IGRA in other forms of extra pulmonary TB.

The major disadvantage we found in using QFT-G for HIV-TB diagnosis is the high number of QFT-G indeterminate results. In order to rule out the technical errors, the stimulated as well as unstimulated plasma samples which were used for QFT-G testing were centrifuged at high speed and measured for IFN-γ level once again using QFT-G ELISA plates. However, none of the indeterminate results became positive. The occurrence of indeterminate results is often pointed out in earlier studies as drawback of IGRA in TB diagnosis especially among individuals with strong immunosuppression [29]. Ferrea *et al* reported that QFT-G showed 20% indeterminate results and it was associated with severity of immunosuppression [30]. In our study, we observed 17% indeterminate results and all the subjects who showed indeterminate results had CD4 count <200 cells/µl. Our observations corroborate Ferrara *et al* study results.

To improve the sensitivity and reduce the indeterminate results of QFT-G, we re-analyzed our data with different cut-off points. Applying the reduced cut-off point suggested by Harada et al, [22] (0.13 IU/ml) for TB antigen specific IFN-γ levels, did not improve the QFT-G sensitivity in our study. When we analyzed the levels of IFN-γ in response to PHA, we found that all the culture confirmed QFT-G indeterminate subjects secreted IFN-γ in the range of 0–0.25 IU/ml and in particular most of the subjects showed ≤0.10 IU/ml.

A valid and lower cut-off point for PHA is required to overcome the misclassification of true negatives as indeterminate for QFT-G, when it is applied for active TB diagnosis in a clinically suspected population. Based on our study results, since the subjects with >0.25 IU/ml of IFN-γ secretion to PHA were able to give valid QFT-G results, we suggest that the IFN-γ level <0.25 IU/ml would be a better cut-off point for PHA. However, this needs further evaluation with a large sample size.

There are some limitations in our study. The diagnosis of active TB in patients with smear and culture negative results, was made only by clinical and radiology based methods and they were not followed up to find their response to anti-TB treatment. Clinical and radiological evidence have the inherent limitation of misclassifying other lung diseases as TB, especially in an endemic setting, which might be one of the reasons for obtaining the low positivity (65%) for QFT-G. However, similar positivity was observed among culture confirmed patients. Thus this limitation is less likely to affect our conclusions. Most of our study patients (73%) had CD4 count <200 cells/µl, which is considered as AIDS defining advanced stage. This might be alternative reason for obtaining low positivity for QFT-G and TST. The previous study which evaluated QFT-G in HIV-TB patients from an endemic country also reported a similar sensitivity (63%) [18]. In addition, another study from India also showed a low QFT-G sensitivity of around 70% in active TB patients [31]. Another limitation of our study is it did not report the specificity of QFT-G in our population since HIV positive TB negative subjects were not recruited. In this study, we used QFT-G and not another commercial IGRA test, T-SPOT. TB. It is reported that T-SPOT. TB is more sensitive than QFT-G [9], [27], [32]. Hence, further studies on the sensitivity of T-SPOT.TB in HIV-TB patients are needed to assess the role of IGRA in finding active TB among suspected cases.

### Conclusions

To conclude, the QFT-G alone or in combination with TST cannot be used to exclude the suspicion of active TB among HIV infected individuals. Considering 65% sensitivity obtained using QFT-G alone, we suggest that the combination of QFT-G with any other test which detects active TB disease in the later stage such as detection of antigen in the urine samples could be better approach to exclude the active TB disease. In addition, the low IFN-γ cut-off point for PHA in indeterminate results, suggested in this study (≤0.25 IU/ml), may improve the proportion of valid QFT-G results, when QFT-G is applied to detect active TB cases among clinically suspected population.
